# Transcriptome profiling of the interconnection of pathways involved in malignant transformation and response to hypoxia

**DOI:** 10.18632/oncotarget.24808

**Published:** 2018-04-13

**Authors:** Frida Danielsson, Erik Fasterius, Devin Sullivan, Linnea Hases, Kemal Sanli, Cheng Zhang, Adil Mardinoglu, Cristina Al-Khalili, Mikael Huss, Mathias Uhlén, Cecilia Williams, Emma Lundberg

**Affiliations:** ^1^ Science for Life Laboratory, School of Biotechnology, KTH, Royal Institute of Technology, Stockholm SE-171 21, Sweden; ^2^ Department of Proteomics, KTH, Royal Institute of Technology, Stockholm SE-106 91, Sweden; ^3^ Department of Biochemistry and Biophysics, Stockholm University, Solna SE-171 21, Sweden; ^4^ Novo Nordisk Foundation Center for Biosustainability, Technical University of Denmark, Hørsholm DK-2970, Denmark; ^5^ Department of Biosciences and Nutrition, Karolinska Institute, Huddinge SE-141 57, Sweden

**Keywords:** hypoxia, transcriptomics, malignant transformation

## Abstract

In tumor tissues, hypoxia is a commonly observed feature resulting from rapidly proliferating cancer cells outgrowing their surrounding vasculature network. Transformed cancer cells are known to exhibit phenotypic alterations, enabling continuous proliferation despite a limited oxygen supply. The four-step isogenic BJ cell model enables studies of defined steps of tumorigenesis: the normal, immortalized, transformed, and metastasizing stages. By transcriptome profiling under atmospheric and moderate hypoxic (3% O_2_) conditions, we observed that despite being highly similar, the four cell lines of the BJ model responded strikingly different to hypoxia. Besides corroborating many of the known responses to hypoxia, we demonstrate that the transcriptome adaptation to moderate hypoxia resembles the process of malignant transformation. The transformed cells displayed a distinct capability of metabolic switching, reflected in reversed gene expression patterns for several genes involved in oxidative phosphorylation and glycolytic pathways. By profiling the stage-specific responses to hypoxia, we identified ASS1 as a potential prognostic marker in hypoxic tumors. This study demonstrates the usefulness of the BJ cell model for highlighting the interconnection of pathways involved in malignant transformation and hypoxic response.

## INTRODUCTION

The transformation of primary cells into malignant counterparts capable of forming tumors is a multistep process where changes in the genome give rise to new characteristics [1]. These include increased rate of proliferation, rewiring of the energy metabolism, increased vascularization, and the ability to escape survival restrictions such as hypoxia [2]. To enable the study of defined steps of tumorigenesis, an isogenic model system for malignant transformation was previously generated in the Weinberg lab [3]. By introducing stepwise genetic changes in the human fibroblast cell line BJ, an important model for molecular studies of cancer was established [3]. The model includes primary cells (normal stage: BJ), ectopic expression of the telomerase catalytic subunit (immortalized stage: BJ hTERT+) followed by stepwise additions of two oncogenes, the Simian virus 40 Large-T (transformed tumor-forming stage: BJ hTERT+ SV40+) and the oncogenic and hyperactive version of H-ras (metastasizing stage: BJ hTERT+ SV40+ RASG12V). Transcriptomic analysis is a well-established approach for understanding gene expression patterns and has been extensively applied in cancer research. Transcriptional changes across various stages during cancer progression have previously been evaluated using genetically modified cell lines or clinical tissue samples of varying degree of malignancy, with data often derived from the Cancer Genome Atlas database (TCGA) [4–10]. Isogenic cell lines have the advantage of enabling evaluation of the effects of specific genetic changes. These models have been critical in the study of cellular mechanisms underlying cancer progression where the effects of key oncogenes can be assessed, for example to compare gene expression between HER2 positive and triple negative breast cancer cells and to assess the effects of various drugs on different isogenic colorectal carcinoma cell lines [11, 12]. Unique to this study is the isogenic nature of the BJ model system that enables hypoxic response to be studied under controlled conditions and allowing for comparison across four specific steps in cancer progression with accumulative genetic changes.

Our previous analysis of the transcriptome changes across this model showed that a functionally diverse set of extracellular proteins was downregulated whereas genes associated with proliferation were upregulated. A number of differentially expressed genes (DEGs) identified across this model, for example BDH1, ANXA1, ANPEP and ANLN, have been corroborated at the protein level in tumor tissues of matching malignancy grades [13]. These data support that the BJ model is a relevant model system for the study of human tumorigenesis and corresponding proliferative capability.

Oxygen is involved in almost all cellular functions. Under normal oxygen conditions, mammalian cells generate the energy that they require for molecular processes through oxidative phosphorylation in the mitochondria. As first demonstrated by Otto Warburg in the 1920s and thereafter confirmed in numerous different studies, transformed cancer cells predominantly generate energy through glycolysis instead of oxidative phosphorylation [14]. This is not universal to all cancer types, but is typically associated with poorly differentiated cancers [14]. The preference for glycolysis applies also under aerobic conditions and regardless of the fact that it is relatively inefficient in terms of ATP generation [15, 16]. This rewiring of energy metabolism is today known as one of the hallmarks of cancer. Further, solid tumors often contain hypoxic regions as a result of the tumor outgrowing the capacity of its surrounding vasculatory network. Creation of such oxygen-deprived microenvironments promotes malignant progression through clonal selection of aggressive phenotypes and subsequent poor prognosis [17]. At a molecular level decreased oxygen levels in tumors are associated with increased hypermethylation events with consequences on gene expression. Promoter regions of genes involved in cell-cycle arrest, DNA repair and apoptosis, glycolysis, metastasis, and angiogenesis have been shown to be targets for hypoxia-induced hypermethylation [18]. This exemplifies how multicellular organisms have evolved strategies for cellular adaptation to the hypoxic environment that is not advantageous for the normal functions of the cell. Hypoxic responses are known to enhance the acquirement of a malignant phenotype [19, 20]; especially through targeting pathways involving the Hypoxia Inducible factor alpha (HIF1a) [21], PI3K/AKT/mTOR [22, 23], MAPK [24, 25] and NFkB [26] that mediate functions that are commonly altered in cancer.

To investigate the transcriptional changes induced by a decreased oxygen supply, and how the response differs between the four isogenically matched cell lines at different degrees of malignancy, we evaluated the transcriptome using RNA sequencing (RNA-seq) after cultivation in both the standard *in vitro* setup of atmospheric oxygen and at moderate hypoxia (3% O_2_) for six passages. By comparing differential gene expression regulation across the model, we explored how the different stages in the BJ cell model respond to moderate hypoxia.

## RESULTS

### Stage-specific responses to moderate hypoxia

**Figure 1:**
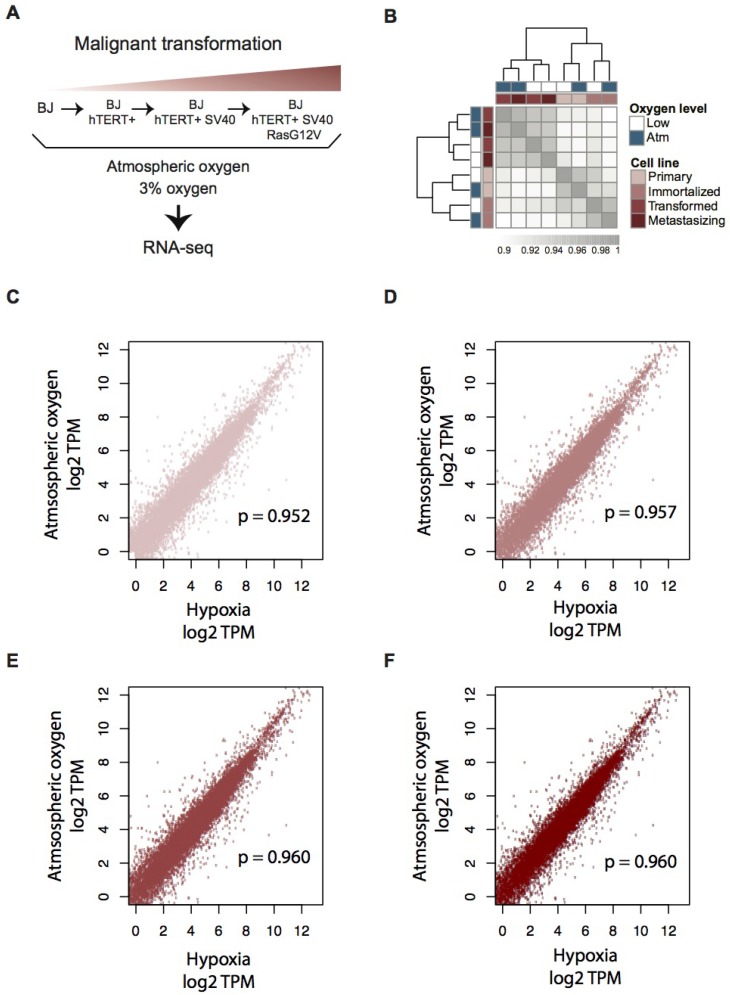
Transcriptome profiling of the four-step BJ cell model for malignant transformation, in atmospheric and moderate hypoxic conditions (**A**) Overview of the BJ cell line model for malignant transformation and experimental workflow. (**B**) Heatmap of pairwise Spearman correlations between RNA expression levels (log_2_ TPM, mean values across biological replicates) for all genes that are expressed (TPM > 1) in at least one sample (*N* = 14,933). Model stage is indicated in red and oxygen state is indicated in blue. Grey scale values indicate Spearman’s correlation coefficients (ρ). (**C**–**F**) Scatter plots of gene expression values (log_2_ TPM) showing the correlation between atmospheric oxygen level versus hypoxic condition. (C) Primary stage, *N* = 13,503, (D) Immortalized stage, *N* = 13,330, (E) Transformed stage, *N* = 13,634, (F) Metastasizing stage, *N* = 13,626). Spearman’s (ρ) correlation coefficients are shown in each plot respectively.

In order to generate a complete overview of the transcriptomic response to hypoxia at different stages of tumorigenesis, we cultured the four stages of the isogenic BJ cell model under atmospheric and 3% oxygen levels, respectively. We corroborated stabilization of HIF1α in response to decreased oxygen (see below) and employed RNA-seq analysis to identify corresponding transcriptomic effects. An overview of the isogenic model system and the experimental setup can be seen in Figure 1A. Cell authentication was performed (Supplementary Figure 1), which confirmed the isogenic nature of the cells and the model’s stage-specific mutations [27]. Gene expression was estimated using the Kallisto software [28], followed by hierarchical clustering of pairwise Spearman correlations for all expressed protein-coding genes across the model (*N* = 14,993). Due to their isogenic nature there were relatively high correlations between gene expression for all the stages and oxygen conditions (Spearman correlation coefficients > 0.9, Figure 1B–1F). These within-model correlations across oxygen levels were notably higher than correlations between different cell lines of related character, such as between immortalized BJ and other immortalized cell lines of both fibroblast origin (HBF TERT88) and mesenchymal stem cell origin (ASC TERT1) (Supplementary Figure 2). Despite this, there was a significant pattern within the model itself: the primary and immortalized samples clustered by model stage whereas the two transformed cell lines clustered according to oxygen level, indicating a higher degree of adaptation to the change in oxygen supply. To investigate this conserved phenotype, differential gene expression was calculated using a cutoff of 0.01 for adjusted *p*-value (Benjamini-Hochberg), a minimum fold change of 2 (log_2_FC > 1), and minimum expression of 1 TPM in at least one of the samples being compared. For each model stage (primary, immortalized, transformed, and metastasizing cells) we identified 909, 1,090, 1,247, and 794 hypoxia-induced differentially expressed genes (hDEGs), respectively (Table 1 and Supplementary Table 1). A total of 3,063 hDEGs, corresponding to about 15% of the protein-coding genome were identified as hDEGs in at least one of the stages. The overlaps between hDEGs for all the stages of the BJ model are presented in Supplementary Figure 3. We note that hypoxic stress caused a strong downregulation of genes in each stage of the model except the third stage (SV40-transformed), where a majority of hDEGs were upregulated (Table 1). These data show that the transcriptional response to oxygen deprivation is more accentuated in the transformed tumor-forming third stage (BJ hTERT+ SV40+), whereas the metastasizing stage has the fewest hDEGs.

**Table 1:** Number of hDEGs and DEGs and their regulation between oxygen conditions and model stages

Between oxygen conditions (hDEGs)				
Regulation	Primary BJ	Immortalized	Transformed	Metastasizing
Up	375	295	778	353
Down	534	795	469	441
Total	909	1090	1247	794
Within model (DEGs, atmospheric oxygen)				
Regulation	Primary versus immortalized	Immortalized versus transformed	Transformed versus metastasizing	
Up	1056	2275	562	
Down	889	1536	404	
Total	1945	3811	966	

### Functional enrichment reveals proliferation and angiogenesis as key responses to hypoxia

**Figure 2:**
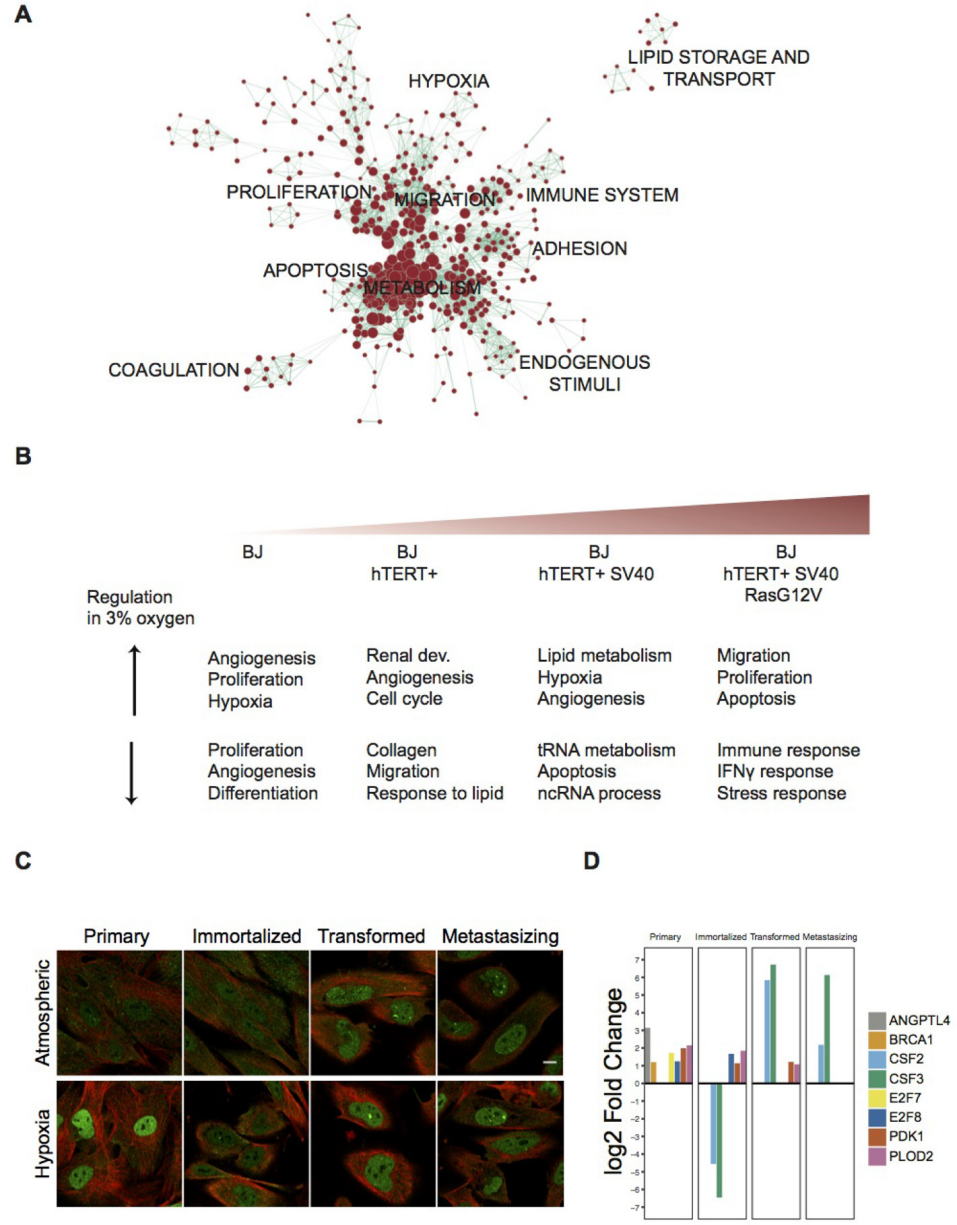
Functional Enrichment Analysis of differentially expressed gene sets and expression of HIF1a across the BJ model in both oxygen conditions (**A**) Enrichment map showing significantly enriched GO terms among all 3,063 hDEGs. Nodes represent gene-sets and edges represent mutual overlap, grouping highly redundant gene-sets together as clusters. Here, node size corresponds to the number of genes within the gene-set and edge thickness corresponds to the number of genes that overlap between two connected gene-sets. Lists of included gene-sets are supplied in Supplementary Table 2. (**B**) Top three significantly enriched clusters based on the Gene Ontology domain Biological Function in DAVID, separated for up- and downregulated genes in hypoxia at each stage of the BJ model. Lists of included gene-sets are supplied in Supplementary Table 2. (**C**) Confocal images of the four cell lines in the model immunofluorescently stained with an antibody (HPA000907) targeting HIF1a in atmospheric oxygen and hypoxia. HIF1a expression is shown in green and microtubules (Tubulin) in red. White scale bar indicates 10 um. (**D**) Bar plot showing log_2_ Fold Change for eight hDEGs involved in enriched functions. Only genes significantly differentially expressed in each stage respectively are included in the plots (*p* < 0.01, log_2_FC > 1, TPM > 1).

In order to deduce which biological functions are modulated upon moderate hypoxia, functional enrichment analysis was performed using the Database for Annotation, Visualization and Integrative Discovery (DAVID) tool [29]. Among the total of 3,063 identified hDEGs, functions related to proliferation, apoptosis, migration, adhesion and metabolic processes were significantly overrepresented (Figure 2A and Supplementary Table 2). Functional annotation clustering analyses for genes differentially up- and downregulated in each cell line respectively, were performed, and the top-three clusters are showed in Figure 2B (see Supplementary Table 2 for all significantly enriched terms and related genes). This analysis revealed that in the non-tumor primary cell line, both up- and downregulated genes are related to angiogenesis and proliferation, whereas angiogenesis and proliferative genes are among the upregulated genes in the malignant stages. Additionally, the top three upregulated clusters in the SV40-transformed cell line are mainly related to lipid metabolism and hypoxia, while the corresponding clusters for the last stage of the model are related to cell migration, proliferation and apoptosis. Corresponding downregulated clusters are related to RNA processing and regulation of apoptosis (third SV40-transformed stage), as well as interferon signaling and stress response (fourth mutated H-Ras stage). This reflects the generalized view provided by functional enrichment analysis, and the complexity of the genetic circuitry affected by the change in oxygen supply. Taken together, the enrichment analyses indicate that the four-stage model not only acquires important cancer-related milestones with decreased degree of differentiation as previously shown [13], but also distinct responses to lower oxygen levels.

### Key roles by NFκB, HIF1A, and interferon signaling pathways

In order to identify the most influential molecular pathways in the response to hypoxia, we analyzed which common regulators were significantly enriched among the regulated genes for each cell line stage (complete results in Supplementary Table 3). By applying subnetwork enrichment analysis on the hDEGs, we could observe a strong response to hypoxia mediated by NFκB, and in particlular TNFα, IL1A, IL1B and IL6-regulated targets, TGFB1, and hypoxia-inducible factor 1-alpha (HIF1A/HIF1α). These were the top overrepresented expression regulators in all four stages (*p* = 3.0 E-57 – 2.0 E-15), and more than 100 hDEGs were associated with each of these factors. Further, we noted a gradual difference in response between the cell lines. While HIF1α was among the top-15 most enriched regulators (*p* = 1.9 E-20) in the first stage, it ranked only below the top-70 most enriched regulators the fourth stage (*p* = 4.6 E-14). The primary cells further exhibited strong responses connected to transcription factors p53, JUN, FGF2 and SP1 (all *p* < 1.0 E-22), while these responses were less predominant in the later stages. In the fourth (mutated H-Ras) stage, interferon gamma (IFNG) took over as the most enriched regulator (*p* = 4.0 E-54, with 188 regulated targets).

### Known hypoxic stress responses corroborated in the model

The most well studied mediator of response to hypoxia is the transcription factor HIF1α. HIF1α mRNA is known to be constitutively expressed in most cells, but under normal oxygen conditions, normoxia [30, 31], HIF1α protein is hydroxylated and targeted for degradation by the von Hippel-Lindau tumor suppressor (pVHL). However, in hypoxia hydroxylation is commonly inhibited enabling HIF1α to escape proteolytic destruction and stabilize [32]. Upregulation of this protein in response to low oxygen conditions is therefore exhibited post-transcriptionally [30, 31]. In our cells, the transcript was expressed in all stages of the model and not significantly regulated (TPM values: 305, 285, 342, 309 across the model stages). We used immunofluorescence to determine whether its protein levels changed across the model’s stages or after hypoxia. In the primary cells (first stage), we detected nuclear expression of HIF1α at low levels at normal oxygen levels, which was accompanied by a strong increase of the protein at hypoxic conditions (Figure 2C, left). Further, we noted a clear increase of HIF1α protein in the later stages of the model (transformed and metastasizing) under atmospheric conditions, with no further increase in response to hypoxia (Figure 2C, right). The observed expression of HIF1α supports that a normal hypoxic response occurs in the primary cells, but that this pathway is modified after transformation. HIF1α has a wide range of transcriptional targets and several of these are upregulated upon hypoxia in the pre-malignant cell lines in our dataset, including the glucose transporter PLAUR, P4HA1, HK2, LDHA and extracellular matrix remodeling PLOD2. We hypothesize that the lower enrichment of HIF1A targets noted in metastasizing cells upon hypoxia as noted above, may be a result of its stabilization (Figure 2D) and activity already in atmospheric oxygen, thus resulting in less changes upon hypoxia. Several other validated genes were also upregulated as a consequence of low oxygen levels. These include PDK1, known to be critical in the adaptation to hypoxia which acts by attenuating mitochondrial ROS production [33, 34]. PDK1 mRNA was significantly upregulated in low oxygen in the three first stages of the BJ model. In the last stage, the metastasizing cells, it was highly expressed already under atmospheric oxygen conditions and did not increase further upon induced hypoxia. PLOD2, a known prognostic factor upregulated in several tumors, and genes involved in angiogenesis, such as E2F, BRCA1 and ANGPTL4, were also among the hypoxia-induced genes in the two pre-malignant cell lines (Figure 2D). Thus, we found that both HIF1a and several other hypoxia-related factors increased normally in the early stages of the model, but in the latter two stages their levels were elevated already under normal oxygen conditions.

### Transcriptome changes in low oxygen resembles the process of malignant transformation

**Figure 3:**
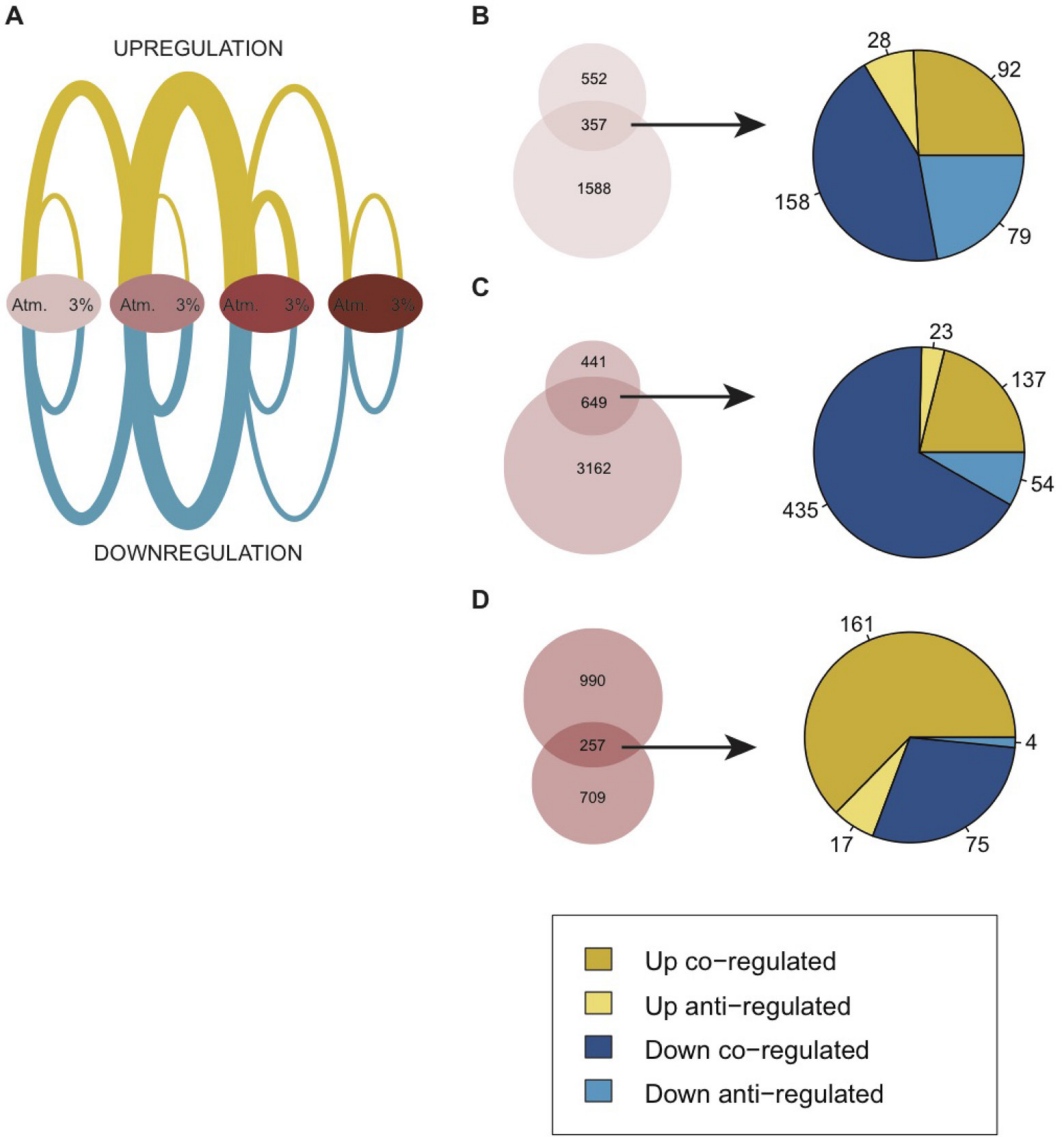
Comparison of differential gene expression due to moderate hypoxia (hDEGs) and differential gene expression changes within the BJ cell model (DEGs) (**A**) Arc plot showing the relative amount of DEGs and hDEGs at each stage of the model. Edges between the ovals represent DEGs and edges within each oval represent hDEGs. Atmospheric oxygen level is abbreviated “Atm.”. Up-regulation is indicated in yellow and down-regulation in blue. Lists of all DEGs and hDEGs are found in Supplementary Tables 1 and 4. (**B**–**D**) Venn diagrams showing the overlap of hDEGs in each stage of the model and DEGs between the current stage and subsequent stage within the model at atmospheric oxygen. Upper circles represent hDEGs and lower circles represent DEGs. Pie charts representing the shared genes in each Venn diagram show the distribution of up- and downregulation and how genes are regulated in the same way (co-regulation) or opposite way (anti-regulation) under hypoxic conditions relative to tumor progression.

Our functional analysis of the identified hDEGs revealed several biological functions that are highly relevant in a cancer context (Figure 2). To connect the changes induced by hypoxia to those related to an increased degree of malignancy within the model, we compared the sets of hDEGs with the corresponding progressive DEGs identified across the model stages (Figure 3A, Table 1, Supplementary Tables 1 and 4).

Of the 909 hDEGs identified in the primary cell line, over a third (*N* = 357) were also differentially expressed between the primary and immortalized stages under atmospheric conditions (under which the model was initially created) and a majority of these (*N* = 250) were regulated in the same direction (co-regulated), mostly downregulated (Figure 3B). Thus, many of the changes incurred by the carcinogenesis process were enhanced by low oxygen. For example, genes involved in cell cycle arrest (such as the Cyclin dependent Kinase inhibitors 1C and 2A [CDKN1C,CDKN2A] and trombospondin 1 and 2 [THBS1,THBS2] were downregulated under both conditions. Others, such as proliferation and vasculature development genes (e.g. E2F receptors and several chemokines) were upregulated in both hypoxia and upon the introduction of hTERT, (Supplementary Tables 1 and 4). For the immortalized cell line the resemblance between hypoxic response and the stepwise process of malignant transformation was even more prominent, sharing 60% (*N* = 649) of the hDEGs with DEGs introduced due to SV40 transformation (Figure 3C). This includes the upregulation of mitotic genes linked to increased proliferation (e.g. the Aurora Kinase [AURKA], Cyclin E2 [CCNE2], kinetochore scaffold 1 [KNL1] and centrosomal protein 83 [CEP83]). Additionally, several matrix metallopeptidases [MMP1, MMP11, MMO14 and MMP2] as well as proteins involved in collagen metabolism [CTSB, CTSK, CTSL, CTSS] and immune responses (interleukins and C-X-C motif chemokine ligands) were downregulated in the immortalized cell line due to both perturbations further supporting a link between hypoxic response and tumor progression (Supplementary Tables 1 and 4).

The third stage, in which an SV40 transformation is introduced and whose gene expression was most affected by the hypoxic condition of all cell lines in the model, displayed the least overlap between hDEGs and DEGs. Only 32% of the hDEGs (*N* = 257) were also altered due to the RasG12V introduction. However, almost the complete set of these genes (*N* = 236) were co-regulated, exhibiting the same direction of regulation upon hypoxia as upon RASG12V introduction. Examples include the upregulation of several hypoxia-linked genes (e.g. BNIP3L, BNIP3, STC1, STC2, VEGFA and ICAM1) (Figure 3D, Supplementary Tables 1 and 4). Thus, in this isogenic cell model we note a strong similarity between the transcriptome regulated by hypoxia and that of the carcinogenesis process.

### SV40 triggers capability of metabolic switching

**Figure 4:**
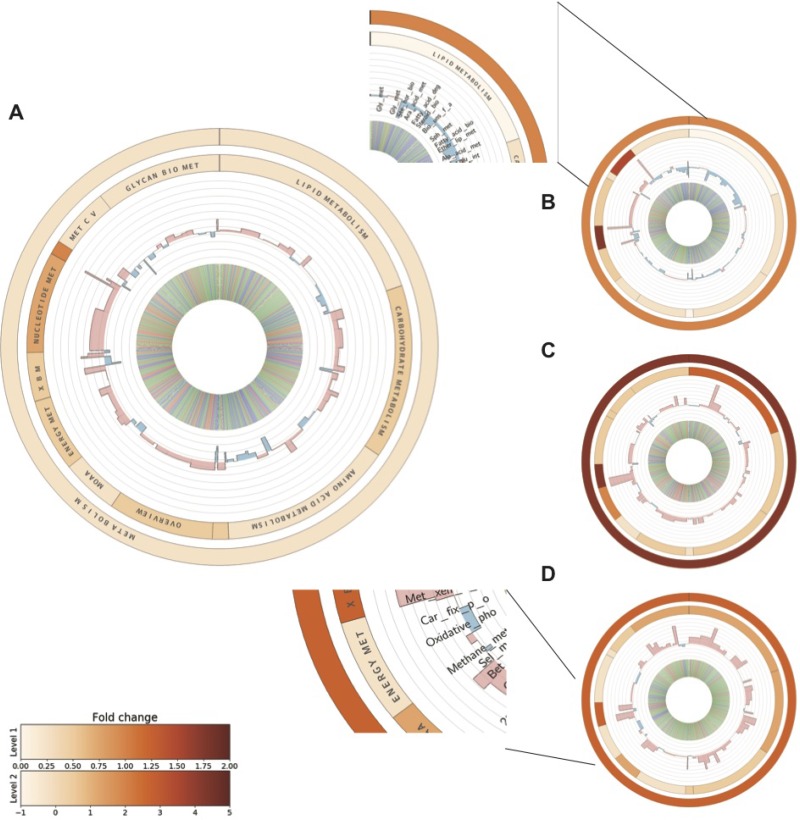
Metabolic profiling of differentially expressed genes at each stage of the BJ model PACFM plots based on the metabolic profiling (using the KEGG Orthology database) of RNA-Seq data showing fold changes of metabolic functions in atmospheric oxygen versus hypoxia for the cell lines across the BJ model: primary stage (**A**), immortalized stage (**B**), transformed stage (**C**) and metastasizing stage (**D**). Functional categories are indicated in the enlarged plot representing the primary stage. The outer and the inner circular heat maps show the average log_2_ fold changes of the functional categories within metabolism and the second hierarchy level KEGG Orthology functions, respectively. The bars in the third level represent the average fold changes of pathway level categories. Upregulated pathways are represented with red, and downregulated pathways are shown in blue. Similarly, the inner most circle shows the fold changes of individual enzymes where red and blue represent up- and downregulation respectively and green bars represent no change in expression between oxygen levels. Full names of the abbreviated KEGG categories can be found in Supplementary Table 5.

Among all hDEGs, enzymes related to metabolic pathways exhibited an overall upregulation (Figure 4A–4D and Supplementary Table 5). Across the different stages of the model, the third SV40-transformed stage displayed a relatively high number of upregulated metabolic genes; supporting our hypothesis that metabolic switching primarily occurs at this stage (Figure 4C). Down-regulation of genes belonging to lipid, carbohydrate and amino acid metabolism occurred in the immortalized cell line (Figure 4B). As shown in Figure 4C–4D, there was an evident upregulation of biosynthetic pathways during low oxygen conditions in the transformed and metastasizing stages. The downregulated enzymes in the immortalized cell line were almost entirely reversed in favor of lipid, carbohydrate and amino acid metabolic pathways in the third transformed stage. This exemplifies how the SV40-transformation appears to induce rewiring of energy metabolism, more specifically fatty acid metabolism, with known implications in clinical cancers [35]. The aldo-keto reductase AKR1B1, involved in glycerolipid metabolism and often overexpressed in human cancers and recently shown to promote breast cancer progression, showed a strong up-regulation in the third SV40-transformed stage (log_2_FC = 2.3), whereas it was downregulated in the immortalized stage (log_2_FC = −2.3) [36]. Stearoyl-CoA (SCD), involved in the metabolism of fatty acids, is another example of a protein that showed a reversed regulation between the immortalized and transformed stages (log_2_FC = −1.8 and log_2_FC = 1.1 respectively). Knockdown of this protein has previously been shown to induce apoptosis in tumor cells grown *in vitro*, indicating the ability for the SV40-transformed cells to evade apoptosis through up-regulation of this metabolic process [37, 38]. Another distinct difference between the stages of the model is the down-regulation of energy metabolism in the metastasizing stage (Figure 4D). In summary, our data support that a metabolic switch takes place in the SV40 transformation stage.

### SV40-transformation renders distinct adaptation to moderate hypoxia

**Figure 5:**
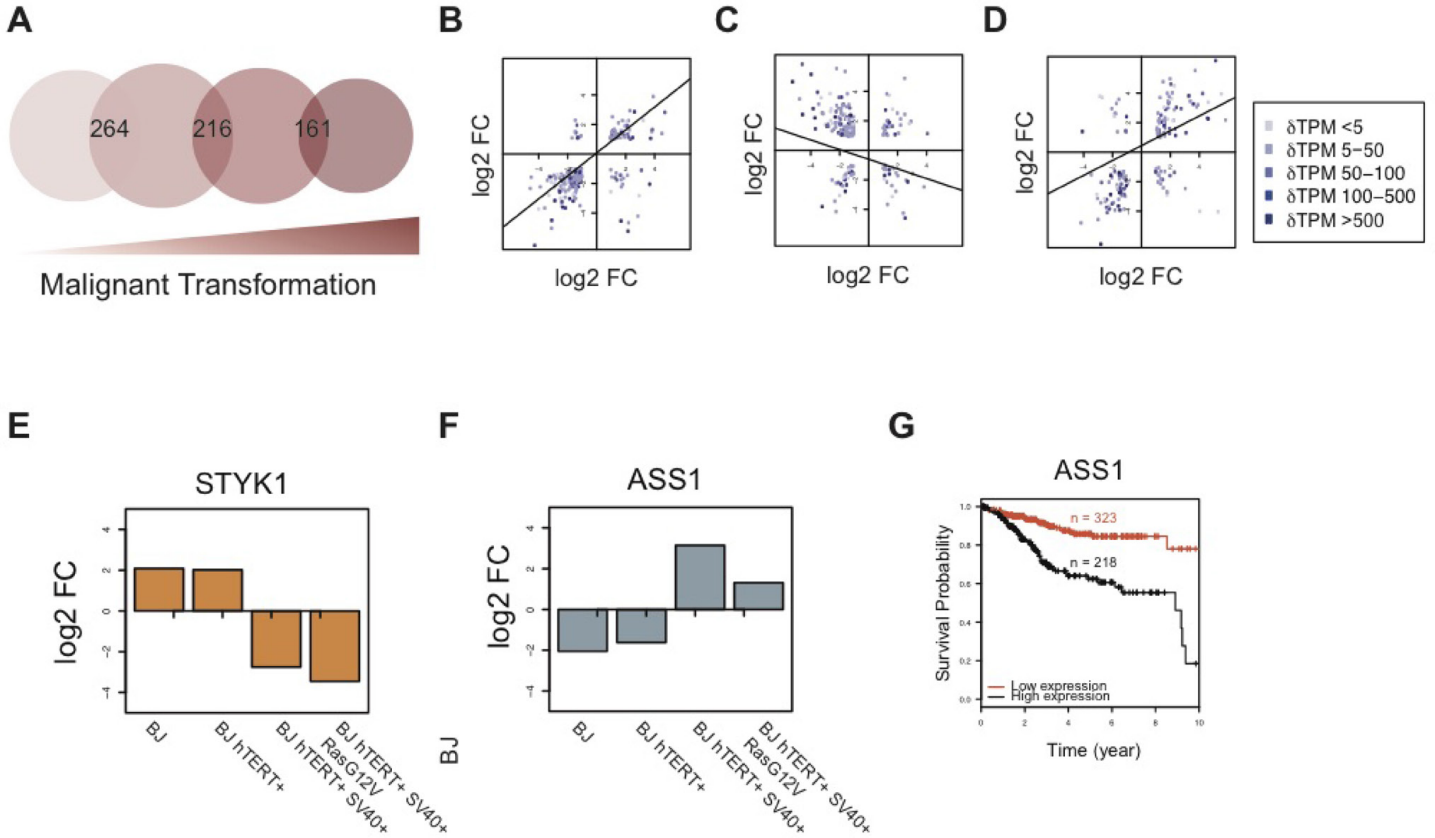
Comparison of hDEGs across the model reveals a shift in response to hypoxia after SV40-transformation (**A**) Venn diagram showing the overlap of hDEGs across sequential tumor progression stages in the BJ model. Circle sizes reflect total number of hDEGs at each stage. Corresponding numbers are found in Table 1. (**B–D**) Comparison of log_2_ fold changes (log_2_FC) among shared hDEGs for each pair of sequential stages is shown for stage 1–2, 2–3, and 3–4 respectively, including lines indicating linear regression. Blue color gradient (light to dark) represents difference between expression values (ΔTPM from low to high). Lists of all hDEGs and their fold changes are found in Supplementary Table 1. (**E**–**F**) The log_2_ fold changes (log_2_FC ) of STYK1 (E) and ASS1 (F) in the four stages of the model, demonstrating the shift in cellular response to hypoxia between pre- and post-transformation of the SV40 oncogene. (**G**) Kaplan–Meier plot for ASS1 including data from an endometrial cancer patient cohort showing survival over a 10 years period. All 541 patients were stratified into two groups with high ASS1 expression (FPKM > 59.6) and low ASS1 expression (FPKM < 59.6), respectively. The FPKM cutoff was optimally selected between 20 and 80 percentiles of the expression of ASS1 in all 541 patients. The separation based on this stratification is significant (*P* = 8.14 × 10^–9^; log rank test), and the hazard ratio is 3.336973. For follow-up on survival until the last event (15 years), see Supplementary Figure 4. Survival data for all patients included are found in Supplementary Table 6.

By comparing hDEGs shared across the model stages, the impact of the SV40 Large-T oncogene becomes clear: not only were the basal levels of HIF1α higher (Figure 2C) and a metabolic switch indicated, but more genes were differentially regulated in response to low oxygen in this stage (stage 3, BJ hTERT+ SV40+) than in the others. Additionally, the hDEGs shared with the previous immortalized stage (BJ hTERT+) were also more often regulated in the opposite direction (anti-regulated). Figure 5A shows the overlap of hDEGs between the sequential steps in the model (for the complete set of overlaps see Supplementary Figure 3). Among the 264 hDEGs that were shared between the primary and the immortalized cells, (Figure 5A) 89% (*N* = 236) were co-regulated (Figure 5B). Several of the upregulated genes are related to proliferation, such as the E2F receptor, MCM10, and the proliferation marker MKI67. In contrast to this, 73% (*n* = 157) of the 216 hDEGs shared between the immortalized and transformed cells were anti-regulated (Figure 5C), indicating a divergent response to hypoxia. Most of these were upregulated in the SV40-transformed cells, but down-regulated in the immortalized cells, such as Interleukin 8 [CXCL8] upregulated in the SV40 transformed cells (log_2_FC = 4.6) but downregulated (log_2_FC = -5.2) in the immortalized cell stage. This protein is known to promote the expression of VEGFA which mediates homeostatic adaptation to hypoxic condition by promoting vascularization to compensate for the decrease in oxygen supply [39]. VEGFA was also upregulated in the SV40 transformed cells (log_2_FC = 2.3). Further, one of the most important regulators of cellular redox state in normal and cancer cells, the mitochondrial superoxide dismutase SOD2, which catalyzes the transformation of superoxide radicals (O_2_-) into either oxygen (O_2_) or peroxide (H_2_O_2_), was anti-regulated in pre-transformed versus transformed stages: downregulated in the immortalized cells (log_2_FC = −3.7) and upregulated in the SV40-transformed cells (log_2_FC = 2.2). The hypoxia-mediated increase of SOD2 in transformed cells points to their ability to evade apoptosis by fighting increased intracellular ROS levels that are triggered by the low oxygen levels. Increased levels of SOD2 have been linked to increased metastatic capability by enabling cancer cells to maintain an increased growth potential and stay protected from excess ROS that would otherwise lead to apoptosis and necrosis [40–42]. The dramatic change due to the SV40-transformation was also apparent when comparing gene expression within the model, rather than between oxygen levels for the same stages. At atmospheric oxygen concentration, under which the model was initially created, we observed 1,945 (primary versus immortalized), 3,811 (immortalized versus transformed), and 966 (transformed versus metastasizing) DEGs respectively. The corresponding numbers at low oxygen levels were 1,595, 2,729, and 395. Interestingly, the stages differed more from each other, in terms of number of DEGs, at atmospheric oxygen levels than in low oxygen. However under both conditions notably fewer genes were differentially expressed between the transformed and metastasizing stages and there were also fewer genes differentially expressed across the final two stages of the model under low oxygen conditions.

Among the 161 hDEGs shared between the transformed and metastasizing stages, a large majority (78% *N*=121) was co-regulated, indicating a conserved response to hypoxia across these stages (Figure 5D). Not surprisingly, HRAS was differently regulated in the HRAS mutated cells, and the mutated variant was upregulated upon low oxygen whereas the WT HRAS in BJ SV40 cells was repressed. Several genes related to lipid metabolism and increased motility were upregulated, for example several chemokines and colony stimulating factors CSF2 and CSF3.

In summary, despite relatively few hDEGs shared across the model, we observed a shared transcriptional response that is more conserved across the primary and immortalized stages, as well as across the transformed and metastasizing stages (Figure 5B–5D). This shift in response between the SV40-transformed stage and the two untransformed stages separates the model system into two groups, supporting the hypothesis that SV40-transformation causes a dramatic divergent shift in cellular response to hypoxia. STYK1 and ASS1, two enzyme-encoding genes, exemplify this shift in response as they were anti-regulated in the two pre-malignant stages compared to the two malignant stages (Figure 5E–5F).

### ASS1 identified as potential marker for poor prognosis in hypoxic tumors

As shown above, the BJ cell model clearly displays a shift in gene expression regulation during hypoxia between the pre-malignant (stages one and two) and malignant stages (stages three and four), more specifically affecting several metabolic pathways. This demonstrates how this model system could potentially be used for the identification of novel prognostic markers with implications in tumor hypoxia. Argininosuccinate synthase (encoded by ASS1) is an enzyme necessary for the biosynthesis of arginine, an amino acid whose abundance has previously been linked to tumor progression induced by hypoxia [43]. Arginine depletion using pegylated arginine deiminase (ADI-PEG20) has been suggested as a therapeutic option for aggressive tumors with hypoxic microenvironments that are hard to treat, currently undergoing clinical trials [43]. ASS1 expression is known to vary across different tumor types and both its loss of expression and overexpression has been shown to influence the effects of therapy [44]. Expression of ASS1 was observed at all stages of the BJ cell model (TPM values 155,679,25,78 across the model under atmospheric oxygen condition). Notably, ASS1 displayed a distinct shift in regulation across oxygen conditions, being over two-fold significantly upregulated in both the transformed and metastasizing cells while over four-fold significantly downregulated in the two pre-malignant stages (Figure 5F).

As tumor hypoxia is considered a major obstacle in clinical oncology due to induced resistance to conventional treatments such as radiotherapy and chemotherapy, we hypothesize that the up-regulation of this protein in malignant cells compared to pre-malignant cells could potentially be related to therapy resistance [45–47]. Survival analysis using clinical data from The Cancer Genome Atlas (TCGA) showed that increased expression of ASS1 implies a significantly worse prognosis with decreased survival probability over a ten-years time period. As shown in Figure 5G, an endometrial cancer patient cohort was stratified into two different sub-groups with high and low expression of ASS1 (cutoff = FPKM 58.6; optimally selected). The sub group with ASS1 expression >58.6 FPKM (“high expression”) exhibited a survival probability less than 20% over a ten-year period, while the other group (“low expression”) exhibited a significantly and dramatically higher survival probability around 80% (*P* = 8.14 × 10–9; log rank test). We speculate that this prognostic value can potentially be linked to hypoxic features of the tumor samples included, for which data is unfortunately lacking. The ASS1 regulation observed in the model at low oxygen exemplifies how the BJ cell model can be used for discovery of prognostic markers, not only for malignant transformation itself but also interconnecting the stage-specific features with induced perturbations.

## DISCUSSION

By profiling transcriptome changes under atmospheric and moderate hypoxic (3% oxygen) conditions, we observed that even though the four cell lines in the BJ model for malignant transformation are highly similar, they display striking differences in their response to decreased oxygen supply. We could trace these differences to the SV40 Large-T transformation step. This oncogene has a large effect on both the steady-state RNA expression of the cells, and on the response to moderate hypoxia. The addition of the mutated HRAS, on the other hand, has a relatively small effect on both states. This separation of the BJ model system into two groups, representing pre- and post-transformation states, is in accordance with our previous findings [13].

The hypoxic environment is capable of inducing cellular signaling with implications in hallmark functions such as proliferation, apoptosis, migration and metabolism that are typically altered during cancer progression. The ability of hypoxia to induce molecular changes that promote the malignant phenotype is clearly consistent with our finding that the response to moderate hypoxia resembles the process of malignant transformation across the BJ model, especially evident for the immortalized stage for which 60% of the identified hDEGs are co-regulated during SV40 transformation. Due to the isogenic and accumulative nature of the model system used in this study, our dataset enables co-regulated responses to be linked to specific events in cancer progression that are represented by the different stages of the model.

The overall up-regulation of the enzymes belonging to metabolic pathways throughout the different stages of cancer cell lines indicates that moderate hypoxia triggers metabolic activity. Distinct upregulation of lipid, carbohydrate and amino acid metabolic pathways in the transformed tumor-forming state in comparison to the previous model stages supports the suggested mechanism that the cells increase the consumption of ATP to trigger elevated fluxes of glycolytic pathways [48]. Our data support that the metastasizing cells expand the ATP consumption to other biosynthetic pathways, including glycan biosynthesis and the metabolism of other amino acids and also increase in the carbohydrate metabolism (Figure 4D). The downregulation of oxidative phosphorylation in post-transformed cells during decreased oxygen supply indicates an adaptation capability of these cells to anaerobic conditions that is not observed in the pre-malignant stages. Oxygen concentrations in human tumors are highly heterogeneous, often containing regions where the oxygen concentration reaches zero, known as anoxic regions [49]. Even though tumor hypoxia often manifests itself at lower oxygen concentrations than used in this study, we observe large differences in gene expression upon moderate hypoxia.

The BJ model has inherent limitations in its ability to mimic the *in vivo* response to hypoxia, above all the fact that cell lines can only serve to approximate the characteristics of cells present within the more complex *in vivo* environment where interactions between molecules and different cell types are important. The fibroblast origin of this model and that it was created under atmospheric oxygen pressure are other limitations. However, as shown before [13], this model remains suitable for studying the mechanisms underlying malignant transformation and provides a unique isogenic system of four defined stages. To gain a more in-depth and mechanistic understanding of the actual interactions underpinning the response to hypoxia observed in this study, our generated transcriptome dataset provides a guidance for follow-up studies which should also explore corresponding protein levels. Although studies support that protein levels, in general, can be directly estimated from RNA levels through the use of gene-specific conversion factors [50], this should be validated under the perturbation of hypoxia.

With this study we demonstrate how pathways involved in malignant transformation and response to hypoxia are interconnected. This is just one example of how the BJ cell model can be exposed to perturbations in order to provide deeper understanding of the link between cancerous stage and response to environmental changes, which will add strength to the overall mechanistic understanding of tumor development and behavior. Potentially, this model could also be used for the identification of novel prognostic markers through the separation of responses to perturbations, as demonstrated here by the differential regulation of ASS1 expression between pre-malignant and malignant cells.

## MATERIALS AND METHODS

### Cell cultivation

All four cell lines in the model were cultivated in Dulbecco’s modified Eagle’s Medium (Sigma-Aldrich) supplemented with 10% Fetal Bovine Serum (Sigma-Aldrich) for six passages at 37°C. Cultivation was performed in a humidified atmosphere containing 5% CO_2_ and both under atmospheric oxygen pressure and under what is considered moderate hypoxia (3% oxygen) for six passages. The cells were grown up to 80% confluence and counted with a Scepter 2.0 Cell Counter (Merck Millipore, Billerica, MA, USA). Cells were cultivated in duplicate plates in parallel for each cell line and oxygen condition.

### RNA sequencing

RNA was extracted from the cells using the RNeasy kit (Qiagen), generating high quality total RNA (i.e. RIN > 8) that was used as input material for library construction with Illumina TruSeq Stranded mRNA reagents (Illumina). Duplicate samples for each cell line were sequenced on the Illumina HiSeq2500 platform.

Raw sequences were mapped to the Human reference genome GrCh37 and further quantified using the Kallisto software [28] to generate normalized Transcript Per Million (TPM) values. TPM values for genes were generated by summing up TPM values for the corresponding transcripts generated by Kallisto. Genes with a TPM value greater than 1 were considered as expressed. Differential expression analysis was performed with the Kallisto software (v0.42.1)+ [28] on the raw FASTQ files with default parameters, followed by differential expression analysis using the TXimport (v1.0.3) [51] and edgeR (v3.14.0)+ [52] R packages. All hDEGs and DEGs within the model are presented in Supplementary Table 4. The GRCh37 human reference genome assembly was used in all steps of the analyses. The generated data discussed in this publication have been deposited in NCBI’s Gene Expression Omnibus [53] and are accessible through GEO Series accession number GSE109367 (https://www.ncbi.nlm.nih.gov/geo/query/acc.cgi?acc=GSE109367).

Functional enrichment analysis was performed using the Database for Annotation, Visualization and Integrative Discovery (DAVID) tool [29], including only the summarized version of the Gene Ontology domain Biological Function, GOTERM_BP_FAT. This was performed against a background of all human genes and medium classification stringency (default). For visualization of the enrichment analysis the application Enrichment map [54] was used for the program Cytoscape [55]. Enriched sub-networks were identified using Pathway Studio’s Expression regulatory sub-network enrichment, Elsevier’s Pathway Studio (11.2.5.9) (https://www.elsevier.com/solutions/pathway-studio-biological-research).

### Immunofluorescence staining of HIF1a

Cells were allowed to attach over night (at 37°C and 5% CO_2_) in a 96-well glass bottom plate (Greiner, Kremsmuenster, Austria) coated with fibronectin (VWR, US) at 12.5 μg/ml in PBS. Subsequent procedures for fixation, permeabilization and Immunostaining of cells have been described elsewhere [56–58]. The HIF1a protein was stained using the antibody HPA000907 that has been validated by overlapping staining in a transgenic HeLa cell line with GFP tagged target protein (published on www.proteinatlas.org). Images were manually acquired using a Leica SP5 laser scanning confocal microscope (DM6000CS) equipped with a 63× HCX PL APO 1.40 oil CS objective (Leica Microsystems, Mannheim, Germany) and connected to the software LAS AF (LAS AF 2.6.0 BETA build 6964, Leica Microsystems). Images were acquired in four sequential steps with the following scanning settings: 16 bit, 600 Hz, line average 2, pixel size 0.08 μm. All images were acquired using the same detector gain (495) in all four cell lines, adjusted based on the strongest staining. Images were colored and assembled as RGB with the software ImageJ 1.46r (National Institutes of Health, USA).

### Cell authentication

Cell line authenticity and mutational analysis was performed as previously described [27]. Briefly, the raw RNA-seq data was aligned using the 2-pass method of the STAR (v2.5.1b) aligner [59], followed by de-duplication, re-calibration and variant calling with the Genome Analysis Toolkit (GATK) Best Practices workflow (v3.5.0) [60]. The resulting variant calls were annotated using SnpEff and SnpSift (v4.2) followed by filtering and analysis using in-house Python and R scripts [61, 62].

### Metabolic profiling

Metabolic profiling of the BJ model was elaborated by following a top-down analysis approach within the broad category of metabolism in the KEGG Orthology database [63]. The program PACFM (v.0.2) was used to plot the log_2_ fold change (log_2_FC) data between the atmospheric and hypoxic treatments of the cell lines at different hierarchy levels within metabolism. In order to facilitate intuitive comparison between the different stages of the BJ model, data was plotted by preserving the absent functional categories in each cancer cell line (Supplementary Table 5).

### Survival analysis for ASS1

Cancer patient samples (*n* = 541) used for survival analysis were collected from Genomic Data Commons (GDC) on June 6, 2016, and all samples used are listed in (Supplementary Table 6). Based on the FPKM values of ASS1, the patients were classified into two groups and their prognoses were examined. All FPKM values for ASS1 from the 20th to 80th percentiles were tested and the value resulting in the lowest log-rank *P* value was selected. The Hazard Ratio was calculated based on COX regression coefficient.

## SUPPLEMENTARY MATERIALS FIGURES AND TABLES














