# Supplementary material for: Transcriptome profiling of the interconnection of pathways involved in malignant transformation and response to hypoxia
Source: Oncotarget. 2018 Apr 13;9(28):19730–44. doi: 10.18632/oncotarget.24808 (PMC5929421; doi:10.18632/oncotarget.24808)
Supplement: Supplementary file 1 [file oncotarget-09-19730-s001.pdf]

Transcriptome profiling of the interconnection of pathways involved in malignant transformation and response to hypoxia

SUPPLEMENTARY MATERIALS

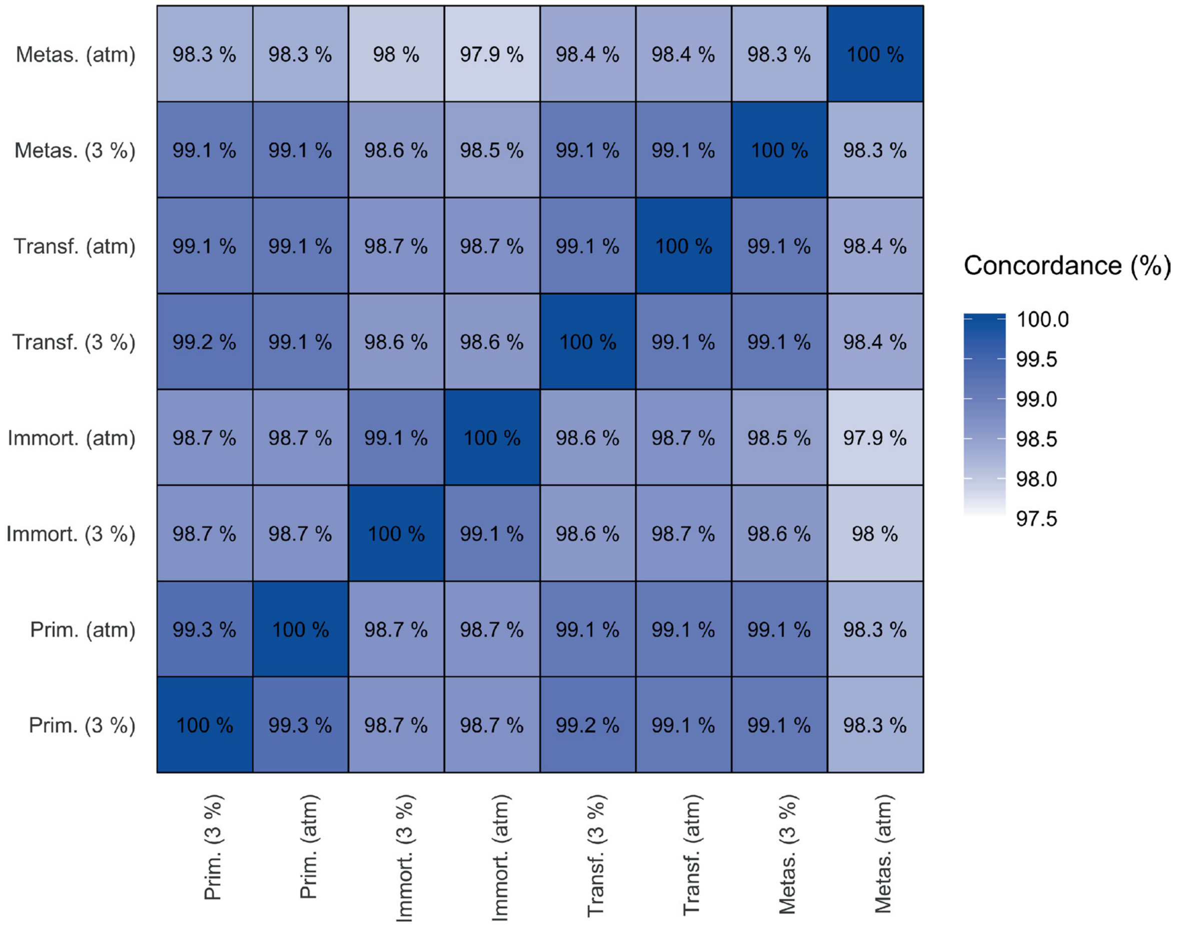

Supplementary Figure 1: Transcriptome-wide, pairwise cell authentication for all the samples included in the study, with the concordance (i.e. proportion of matching variants between two arbitrary samples) as a color gradient of increasing similarity. A previously used threshold for authentic cell lines is 90% concordance, which is achieved for all cells in this study.

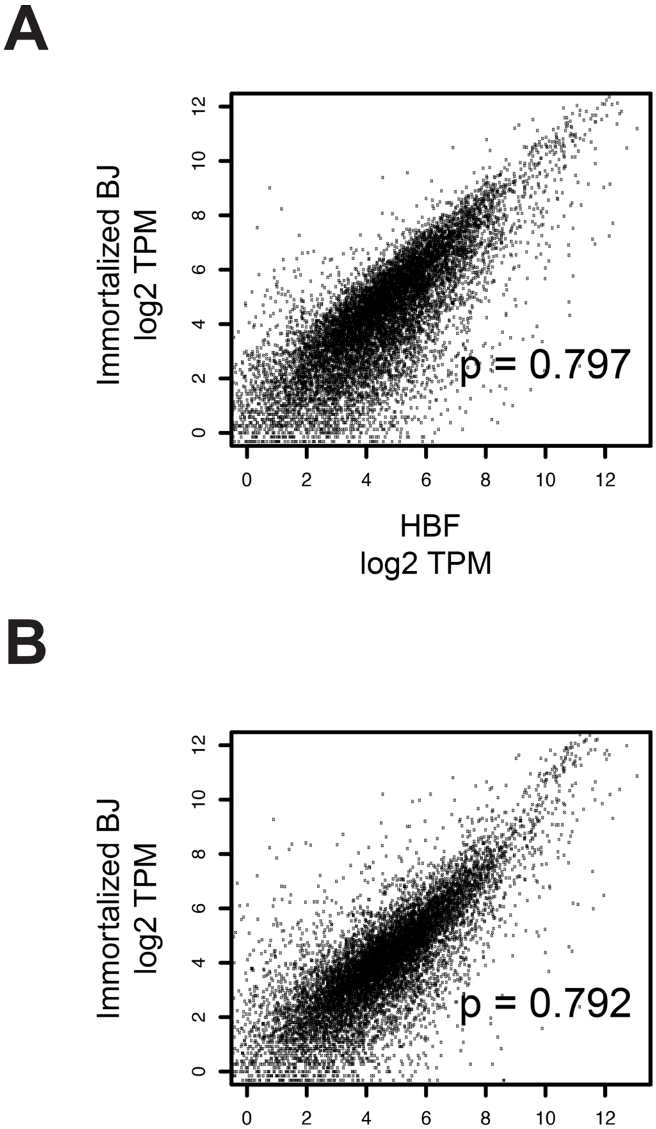

Supplementary Figure 2: (**A–B**) Scatter plots of gene expression values (log2 TPM) showing the correlation between the hTERT-immortalized BJ cell line and two hTERT-immortalized cell lines of different origin (A) HBF, N = 11,871 and (B) ASC, N = 11,871). Spearman's (ρ) correlation coefficients are shown in each plot respectively.

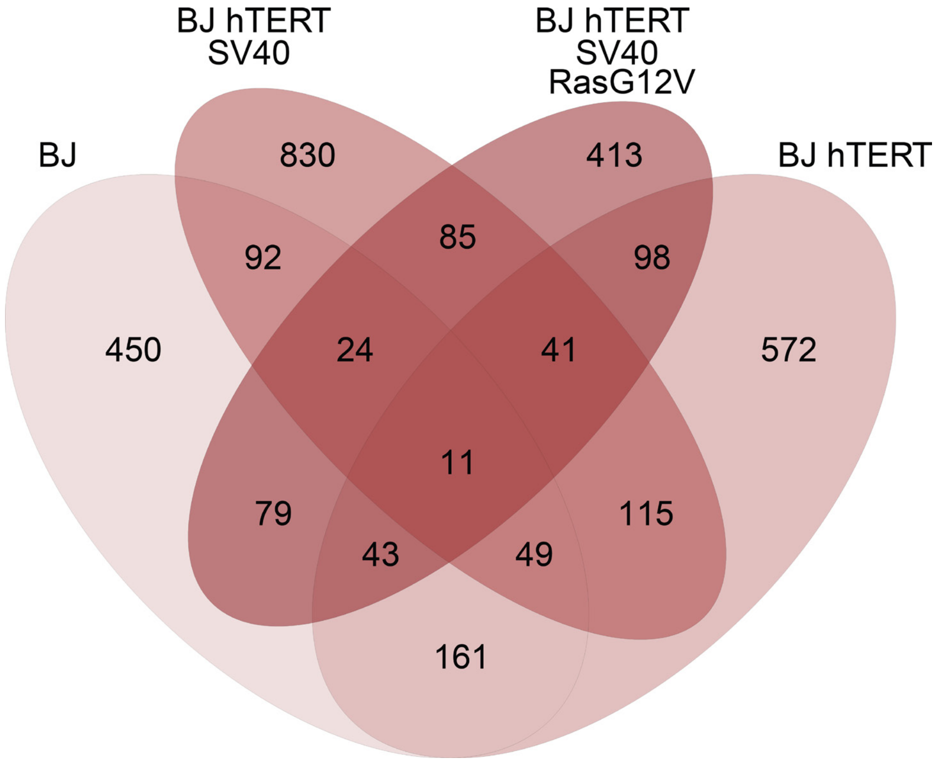

Supplementary Figure 3: Venn diagram showing the overlap of hDEGs between all stages of the BJ cell model.

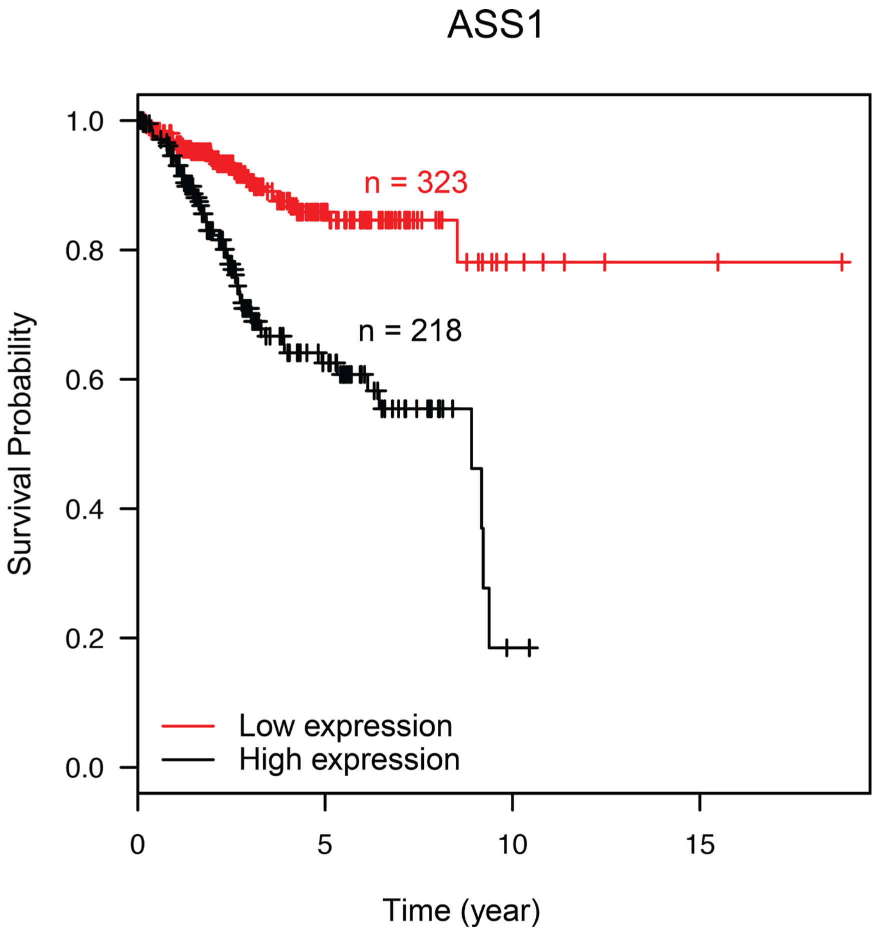

Supplementary Figure 4: Kaplan-Meier plot for ASS1 including data from an endometrial cancer patient cohort, showing survival probability over a 15 years time-period. All 541 patients were stratified into two groups with high ASS1 expression (FPKM > 59.6) and low ASS1 expression (FPKM < 59.6), respectively. The FPKM cutoff was optimally selected between 20 and 80 percentiles of the expression of ASS1 in all 541 patients. The separation based on this stratification is significant ($P = 8.14 \times 10^{-9}$; log rank test), and the hazard ratio is 3.336973.

Supplementary Table 1: Containing all identified hDEGs in the four stages respectively, presented in four different sheets. See Supplementary_Table_1

Supplementary Table 2: Containing results from DAVID analysis used in Figure 2. In the first sheet, enriched terms for all identified hDEGs in total are identified, and the following sheets. See Supplementary_Table_2

Supplementary Table 3: Containing results from subnetwork analysis of hDEGs in each cell line of the model respectively, presented in different sheets. See Supplementary_Table_3

Supplementary Table 4: Containing all identified DEGs in the three sequential transitions across the model stages, presented in three different sheets. See Supplementary_Table_4

Supplementary Table 5: Related to Figure 4. See Supplementary_Table_5

Supplementary Table 6: Containing patient survival data for ASS1 derived from the Cancer Genome Atlas (TCGA). See Supplementary_Table_6
